# Autologous stem cell transplantation in two children with disabling pansclerotic morphea

**DOI:** 10.1186/1546-0096-9-S1-P77

**Published:** 2011-09-14

**Authors:** M Moll, U Holzer, C Zimmer, N Rieber, JB Kuemmerle-Deschner

**Affiliations:** 1 Department of Rheumatology, General Pediatrics, Hematology and Oncology, University children`s hospital, Tübingen, Germany

## Background

Disabling pansclerotic morphea is an extremely rare and severe disorder in children, systemic treatment with corticosteroids and methotrexate (MTX) or mycophenolate mofetil (MMF) are the most common therapies. However, patients can develop severe disabilities. Autologous stem cell transplantion (ASCT) is a successful treatment option for systemic scleroderma and might also be beneficial for severe therapy resistant disabling pansclerotic morphea.

## Aim

To report about two children with severe disabling morphea, who received ASCT.

## Methods

Two children were diagnosed at the age of six years with disabling pansclerotic morphea of the right (patient 1, male) and left (patient 2, female) leg and trunk, respectively. In spite of therapy with PUVA, pulsed methylprednisolone, MTX and MMF (patient 1) disease progression was rapid in both children. ASCT was performed using a CD3/CD19-depleted graft after immunoablative conditioning with fludarabine, cyclophosphamide and anti-thymocyte globuline at 16 months (patient 1) and 11 months (patient 2) after initial diagnosis.

## Patients and results

Progressions of disabling morphea was halted in both children. The skin softened and the leg function improved and no immunosuppressive therapy was needed , after uneventful ASCT. However, at 18 months following ASCT, patient 1 developed new cutaneus but no deep or disabling lesions and glucocorticoid and MTX treatment was restarted. At 12 months following ASCT, patient 2 developed new cutaneus, osseus, cerebral and renal lesions. She received MTX and corticosteroids for 4 months until her parents refused further therapy.

## Conclusions

ASCT can stop the rapid progress of the disabling lesions in children with disabling morphea. However new skin lesions and other related lesions may reoccur. It remains unclear, if immunosuppressive therapy immediatedly following ASCT could have induced persistent remission.

